# Impacts of climate change on suitability zonation for potato cultivation in Jilin Province, Northeast China

**DOI:** 10.1038/s41598-021-91273-5

**Published:** 2021-09-06

**Authors:** Yaqiu Zhu, Qiang Yu, Qiyou Luo, Hua Zhang, Jinling Zhao, Zhanghong Ju, Yating Du, Yadong Yang

**Affiliations:** 1 Institute of Agricultural Resources and Regional Planning, Chinese Academy of Agricultural Sciences, Beijing, 100081 China; 2 State Key Laboratory of Vegetation and Environmental Change, Institute of Botany, Chinese Academy of Sciences, Beijing, 100093 China

**Keywords:** Climate-change impacts, Agroecology

## Abstract

Global climate change is causing notable shifts in the environmental suitability of the main regions involved in potato cultivation and has, thus, changed the production potential of potatoes. These shifts can be mapped at fine scales to better understand climate change within areas of potato cultivation and to find infrastructural- and breeding-based solutions. As a case study, we have identified and mapped the structural and spatial shifts that occurred in areas suitable for potato cultivation in Jilin Province, China. We identified a discontinuity in climate change trends between 1961 and 2018 based on data for Jilin Province, and analyzed the averages and linear trends for six important climatic parameters. We used the averages of these climatic parameters to establish climate models for the province and determined cultivation using a multi-criteria, decision-based model that integrates Analytical Hierarchy Process Weighted Principal Component Analysis (AHP-PCA) and Geographic Information System (GIS). We mapped the environmentally suitable areas for potato cultivation at a 3-km resolution based on the geo-climate model for each time period and analyzed differences between them. We found that "Most suitable” areas for potato cultivation were mainly distributed in the central area of Jilin Province, “Suitable” areas were located in the northwestern plains, and “Sub-suitable” areas were located in the eastern mountainous areas. In contrast, “Not suitable” areas occur mainly in the high-altitude areas in the east. The areas of “Most suitable” and “Suitable” areas for potato cultivation in Jilin Province were increasing, with increasing rates of 0.37 × 1,000 km^2^ decade^−1^ (R^2^ = 0.58, *P* < 0.01) and 0.20 × 1,000 km^2^ decade^−1^ (R^2^ = 0.28, *P* < 0.01), respectively, while the extent of “Sub-suitable” areas is decreasing, with a decreasing rate of 0.58 × 1,000 km^2^ decade^−1^ (R^2^ = 0.53, *P* < 0.05). The area of “Not suitable” areas had undergone little change. “Most suitable” and “Suitable” areas for potato cultivation showed a trend towards northward expansion. Overall, our results suggest that global climate change has had a positive impact on potato cultivation in Jilin Province over the past 58 years.

## Introduction

The global surface temperature has increased by 0.85 °C in the past 130 years, especially in the high latitudes of the Northern Hemisphere^1^. As with other areas of the world, the temperature of Jilin Province has experienced an increase for the last six decades^2,3^. Jilin is located in the Northeast China, and its latitude makes it relatively highly susceptible to the effects of climate change within the temperate latitudes^4^. Jilin Province is one of the main potato-producing regions in China^5^. The province is rich in arable land resources and has a climate and geographical environment highly conducive to the growth and development of potatoes. Within Jilin Province, long-term and high spatial resolution research on potato cultivation provides a globally and nationally critical reference that is significant for promoting economic growth and development within the potato industry.

In general, higher temperatures increase the rate of crop growth and development, which influence yield^6–8^, and climate change leads to shifts in the suitable geographic ranges for the growth of specific crops and cultivars^9,10^. Moreover, in some cases, the phenology of crop plants is changing within the areas where they are cultivated, leading to temporal shifts in production with major economic impacts^11,12^. Combined, these two factors can lead to major changes to crop productivity within municipalities and broader regions, such as those already observed for soybean production and rice cultivation^13,14^. In turn, this affects local, regional, and global food availability and security^15^. Global climate change has shifted the climatic suitability for crops in a region, particularly in the northern high latitudes^16–18^, which should not be overlooked by farmers.

Globally, potato (*Solanum tuberosum* L.) is the fourth most widely cultivated crop after maize, rice, and wheat^19^, with more than 91.9 million tons produced annually across an area of about 4,789.5 thousand hectares, with average yield of 19.1 t ha^-1^ in China^20^. In 2015, China launched the potato staple food strategy, which acknowledged and facilitated the role of potatoes in maintaining food security^21,22^. However, potato cultivation faces possible challenges due to ongoing anthropogenic climate change^23–28^, which is impacting many agricultural systems^29–31^. Several studies predicted an overall decrease in potato yield under the effect of global climate change in Eastern Europe and northern America^32,33^. However, higher temperatures in England and Wales^34^, southern Brazil^35^, and within the mid-latitudes and tropical highlands^33,36^ are predicted to benefit potato yield. Thus, the present understanding of the impacts of climate change on potato yield appears to vary to opposite extremes by regions.

Ecological suitability is defined as the degree of agreement to which actual temperature, light, water, soil, and other climatic conditions satisfy the requirements of crop growth without considering other limiting factors. A high degree of agreement means strong suitability and good crop growth and development^16,37^. Studies on suitability could offer scientific evidence to reflect yield, quality, and layout of crops, as well as to promote the crop distribution under the effect of climate change^38–40^. Therefore, to obtain information on geographic shifts of suitable areas for crop cultivation under climatic conditions is the starting point for adaptation planning in agriculture^41^.

Previous case studies on potato cultivation involve breeding cultivar improvement, agro-technique development^42,43^, field experiment, and potato yield estimation^44–46^. Conventional research^47,48^ have focused on the development of potato industry. However, to our knowledge, no systematic study on the estimation of the variation of ecologically suitable areas under the influence of climate change has been reported for potato cultivation in Jilin Province.

The aim of this study was to identify shifts occurring in the spatial distribution of ecologically suitable areas for potato cultivation within Jilin Province, China between 1961 and 2018 under the influence of climate change. Here, the inter-annual variations of climatic factors were analyzed, and temporal and spatial distributions of suitable areas were estimated for climate change impacts across Jilin Province using gridded and point-based historical climate datasets. The map for each criterion was prepared using Geographic Information System (GIS) with weight values obtained from a widely used evaluation model Analytical Hierarchy Process Weighted Principal Component Analysis (AHP-PCA). These maps were combined to generate suitability maps for potato cultivation by using comprehensive ecological suitability indices. We reported the agro-climato-edaphic zonation for potato cultivation in Jilin Province. During the analysis, the soil factors were constant, and only the influence of climatic factors on changes in suitable areas was considered. The objectives of this study were to: (i) characterize the trends for six climatic factors between 1961 and 2018; (ii) analyze the variation of spatial distribution of climate inclination rates across the study area from 1961 to 2018; (iii) identify shifts occurring in the temporal and spatial distributions of suitable areas for potato cultivation in Jilin Province between 1961 and 2018; (iv) analyze trends in the climate-driven suitability zonation across Jilin Province; and (v) explore changes in the area of suitability zonation for potato cultivation in Jilin Province between 1961 and 2018.

## Materials and methods

### Study area

**Figure 1:**
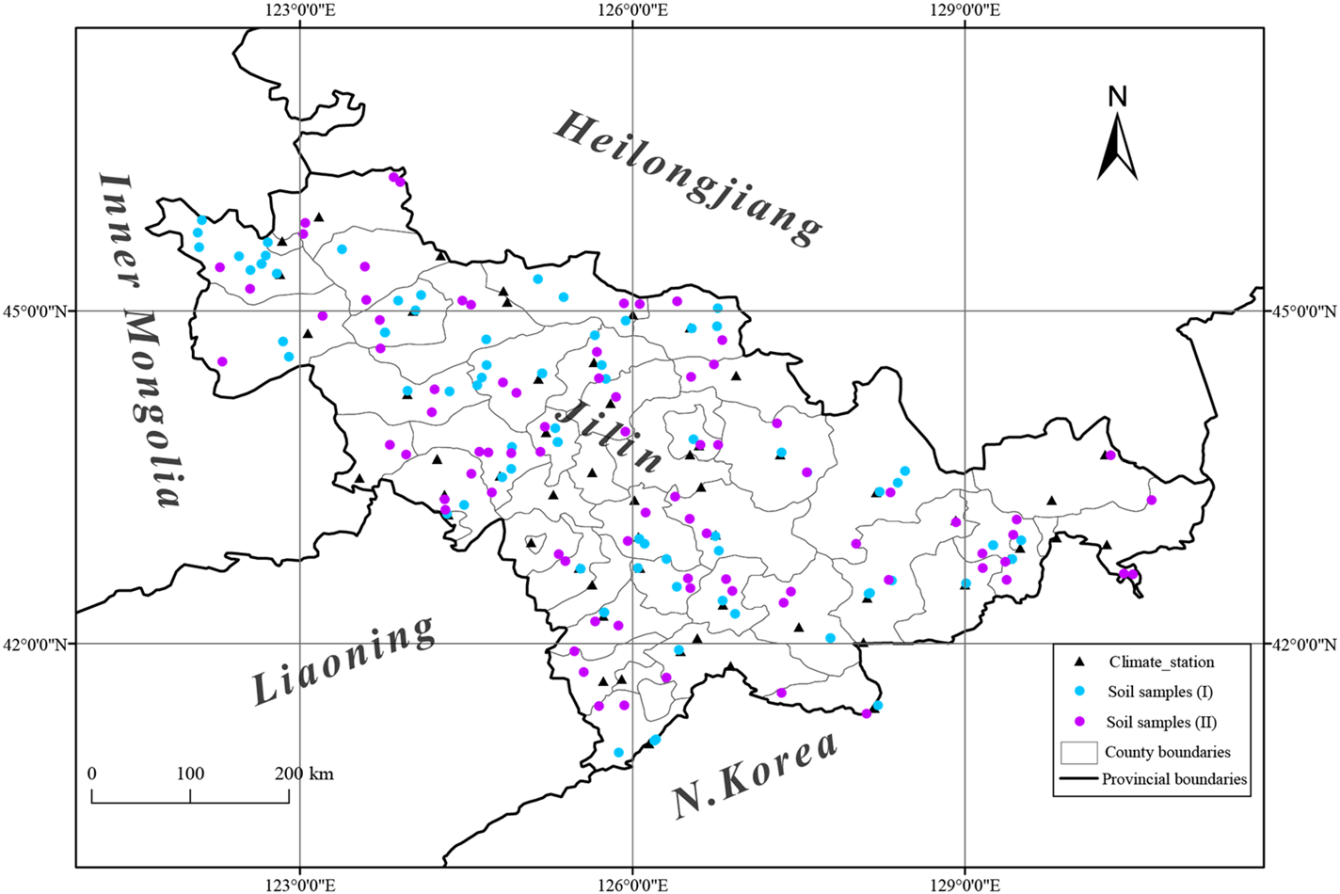
Spatial distribution of 51 meteorological stations and soil sampling sites in the study area. Soil data were divided into two categories. Soil samples (I): soil mechanical compositions, involving 81 sampling points; soil samples (II): soil physico-chemical properties, involving 79 sampling points. The map was created using ArcGIS v. 10.4.1 (http://www.esri.com/software/arcgis).

This study was conducted in Jilin Province, which is located in the center of Northeast China (40°52ʹ N–46°18ʹ N, 121°38′ E–131°19ʹ E) and covers an area of approximately 187,400 km^2^, with an elevation varying from 5 m to 2,691 m (Fig. 1). The study area has a temperate continental monsoon climate and is climatologically humid, semi-humid, and semi-arid from the southeast to the northwest. The annual mean temperature and annual total precipitation form a southeast-northwest gradient; the eastern part is relatively humid and rainy while the western region is dry in the summer months. Generally, 70–80% of the annual precipitation occurs from June to September, with the most abundant rainfall in the east. The long-term average annual temperature and average annual rainfall are 5.8 °C and 687.0 mm, respectively^49^. Crop cultivation is mostly concentrated in the black soil region^50^. The soil types of cultivated lands mainly include black soil, sand, and paddy soil, which are suitable for potato growth.

Potato growth is highly dependent on temperature and light. Jilin Province, as one of the main potato-producing areas in China, possesses sufficient sunlight and exhibits large temperature difference between day and night. Generally, potato cultivation occurs from April to May, depending on the lowest temperature (5 °C), and potatoes are harvested from August to October of the same year. Among potato production areas, mid-late maturing cultivars (e.g., Yanshu No. 4, Atlantic, Jishu No. 1, and Summer) account for about 70%, while early maturing cultivars (e.g., Favorita, Youjin, and Fujin) account for 30%^51^.

### Data

#### Climate data

Climate data were obtained from the National Meteorological Information Center, China Meteorological Administration (http://data.cma.cn), including 51 national standard meteorological stations in Jilin Province (Fig. 1). The meteorological data contain daily average temperature, daily maximum temperature, daily minimum temperature, daily sunshine hours, and daily precipitation during 1957–2018. Based on the periods of potato sowing and harvesting in Jilin Province, the climate data between April 1 and September 31 each year were selected. To avoid the impact of extreme weather within a single year on the inter-annual climate change, we used 5-year moving average values of climate data rather than single-year values to establish a geo-climate model using regression analysis and evaluated changes in suitable areas for potato cultivation under the influence of climate change.

#### Topography data

Topography data were extracted from the digital elevation model (DEM) sourced from the geospatial data cloud SRTM (http://www.gscloud.cn). Through a series of processes such as adding X–Y axis, splicing, vector data layering, filtering, cropping, and resampling of raster data on the ArcGIS platform, digitized elevation model (90-m resolution) maps were used to derivate layers such as longitude, latitude, slope, and aspect (Fig. 1).

#### Soil data

Soil mechanical composition data (81 sampling sites) were extracted from the National Science and Technology Infrastructure Platform (http://soil.geodata.cn) and soil physico-chemical property data (79 sampling sites) were provided by the Soil and Fertilizer General Station of Jilin Province (http://www.jltf.cn). The sequence number of the occurrence layer is 1, and the thickness is about 20-50 cm. The soil properties extracted included contents of soil sand, silt, and clay, pH, and contents of nutrients such as organic matter (OM), quick-acting potassium (QAK), available nitrogen (AN), and available phosphorus (AP) (Fig. 1; Tables S1-S2).

The soil data were rasterized using kriging. First, the soil mechanical composition data were converted into spherical coordinates, and then ordinary Kriging interpolation was used to spatialize the soil mechanical composition data. co-kriging was used to interpolate spatialize the soil physico-chemical property data. Due to limited soil samples and the lack of a continuous dataset in the study area, the soil data in 2018 were selected as a fixed background for the analysis.

### Analysis of climatic factors

We used six climatic factors in this study. Usually, potatoes have has different requirements for light, heat, and water in each growth and development stage. We used average daily temperature during the growth period (ADT/°C, mean of daily average temperature from April 1st to September 30th) and active accumulated temperature ≥ 10 °C (AAT/°C d, sum of active accumulated temperature ≥ 10 °C from April 1st to September 30th) from 1961 to 2018 to reflect the temperature conditions of potato growth^52–56^. ADT at 14–17 °C was evaluated as “Most suitable”; 10–14 °C or 17–20 °C as “Suitable”; 8–10 °C or 20–24 °C as “Sub-suitable”; < 17 °C or > 24 °C as “Not suitable” for potato growth in the study area. AAT for mid-late maturing varieties at 2000–3000 °Cd was evaluated as “Most suitable”; 1,500–2,000 °Cd or 3,000–6,000 °Cd as “Suitable”; 1,300–1,500 °Cd or 6,000–8,000 °Cd as “Sub-suitable”; < 1,300 °Cd or > 8,000 °Cd as “Not suitable”.

The average temperature in July (ATJ, mean of daily average temperature in July) and the day/night temperature difference from July to August (DIF/°C, mean of the day/night temperature difference from July 1st to August 31st) are the key climatic factors for the expansion of potato chunks, which have significant correlation with the meteorological yield of potato 53–57. ATJ at 16–20 °C was evaluated as “Most suitable”; 15–16 °C or 20–24 °C as “Suitable”; 12–15 °C or 24–28 °C as “Sub-suitable”; < 10 °C or > 28 °C as “Not suitable”. DIF at 8–12 °C was evaluated as “Most suitable”; 5–8 °C as “Suitable”; 2–5 °C as “Sub-suitable”; < 2 °C as “Not suitable” in the study area.

During the growth and development of potato, there is a great demand for water, especially from the budding stage to the swelling stage of potato growth, which are extremely sensitive to water supply^52–56^. The total precipitation during the growth period (PP/mm, sum of the daily precipitation from April 1st to September 30th) at 700–900 mm was evaluated as “Most suitable”; 600–700 mm or 900–1,200 mm as “Suitable”; 500–600 mm or 1,200–1,500 mm as “Sub-suitable”; < 500 mm or > 1,500 mm as “‘Not suitable” in the study area.

Short daylight and appropriate high temperature during the seedling stage are beneficial to promote potato root development, forming strong seedlings and increasing potato formation^52–56^. The total sunshine duration during potato growth (SD/hours, sum of the daily sunshine duration from April 1st to September 30th) at 900–1,200 h was evaluated as “Most suitable”; 700–900 h or 1,200–1,500 h as “Suitable”; 400–700 h or 1,500–1,800 h as “Sub-suitable”; < 400 h or >1,800 h as “Not suitable”.

### Methods

First, climatic factors were simulated using geo-climate models. Then, the AHP-PCA model was employed for suitability evaluation, and the satellite-based gridded environmental data were applied for suitability mapping. Finally, the degree of changes in climatic factors and suitable geographic ranges were calculated. These data were interpolated into the surface grid data with a spatial resolution of 0.03° × 0.03° (~3 km × 3 km)^57,58^. All maps and statistical analyses were generated using ArcGIS 10.4.1^59^ and R 3.6.3^60^.

#### Geo-climate model building

Topographic factors such as longitude, latitude, and altitude dominate the distribution of climate factors, and directly affect the solar radiation budget and atmospheric circulation, which makes the climate resources to demonstrate obvious spatial differences in both vertical and horizontal directions^61,62^. Based on the meteorological data and geographic information of each meteorological station, we established geo-climate models and used them to calculate the climate distribution of the study area. The difference between the highest temperature and the lowest temperature from July 1st to August 31st was used to calculate the grid layer of DIF. The relationship between climate zoning indicators and geographic factors is expressed as follows:

1 $$ F = f\left( {\lambda ,\varphi ,h} \right) + \varepsilon $$

where, *F* is the simulated value of grid point of the climate zoning index; *λ, φ,* and *h* represent longitude (°), latitude (°), and altitude (m), respectively; *f* (*λ,φ,h*) is called climatological equation of regionalization index; and *ε* is the influence of local small topography and random factors on climate (i.e., comprehensive geographical residual term).

Residual correction : Affected by local topography and random factors, the variation of climatic factors is random, which will cause errors in the calculation of geo-climate models. Therefore, the inverse distance weight (IDW) routine in ArcGIS was used to derive the simulated value of the comprehensive geographical residual term *ε* raster^63^. The interpolation calculation formula is:

2 $$\varepsilon ={\sum }_{i=1}^{n}\frac{{\varepsilon }_{i}}{{d}_{i}^{k}}/{\sum }_{i=1}^{n}{d}_{i}^{k}$$

where, *ε* is the simulated value of the grid point of the residual term of climatic factors; $n$ is the number of meteorological stations; ${\varepsilon }_{i}$ is the residual value of the climate factor of the $i$-th meteorological station; ${d}_{i}$ is the Euclidean distance between the grid point and the $i$-th meteorological station; *k* is the power of the distance.

#### AHP-PCA and GIS based suitability analysis for potato cultivation

The suitability map for potato cultivation was generated based on identified criteria that are relevant to the climatic, soil environmental, and geophysical conditions considered. Details of the data analysis procedure, model application, and suitability classification are described as follows.

**Figure 2:**
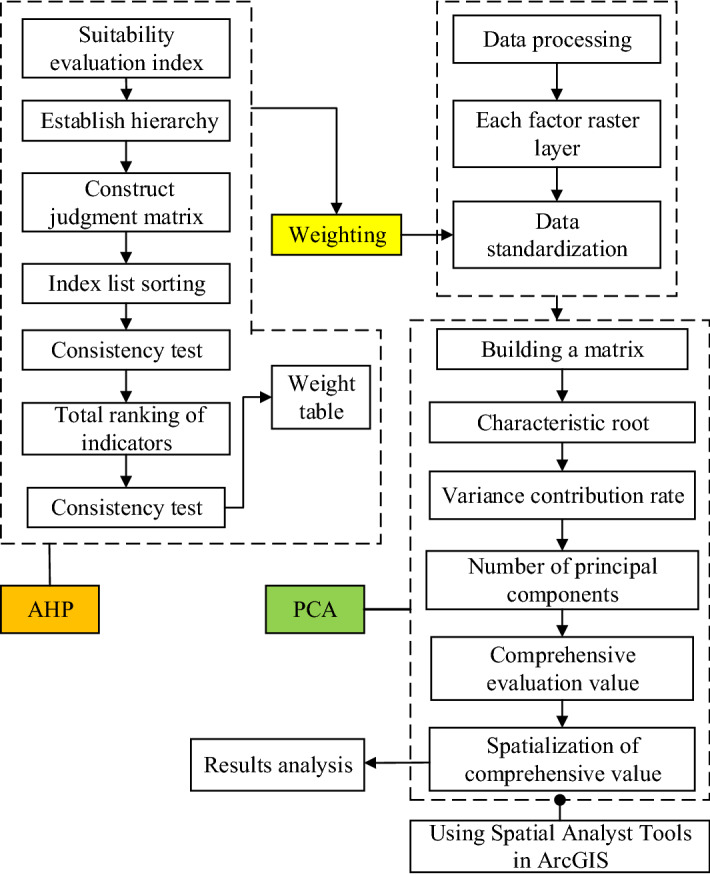
Diagrammatic flow of the Analytical Hierarchy Process (AHP) weighted Principal Component Analysis (PCA) model evaluation process.

- AHP-PCA model
  Analytical Hierarchy Process (AHP) is a multi-criterion decision-based approach developed for analyzing complex decisions involving multiple criteria^38,64,65^. Principal Component Analysis (PCA) is a multivariate statistical data analysis technique that combines all input variables using a linear combination into a number of principal components that retain the most variance within the original data to identify possible patterns or clusters between objects and variables. In this study, we used AHP to calculate the weight of each zoning indicator in the evaluation index system^66,67^, and then, we explored the comprehensive relationship of suitability evaluation factors using the grid calculator and PCA tool on the ArcGIS platform. The first principal component will have the greatest variance, the second will show the second most variance not described by the first, and so forth. In most cases, the first three or four raster bands of the resulting multiband raster from principal components tool will describe more than 95% of the variance, that is, the cumulative contribution rate of the principal component reaches more than 95%. The variance of the weighted original data becomes larger, leading to more scientific and reasonable evaluation results. In summary, the proposed approach is achieved as follows (Fig. 2):
  Step 1: The weight of each index was calculated by using AHP and consistency test;
  Step 2: The indicators were standardized using the *Z-Score* method;
  Step 3: The weights calculated in Step 1 were loaded onto the standardized indicators;
  Step 4: A standardized matrix was built and the correlation coefficient matrix was calculated;
  Step 5: The principal components was filtered and determined;
  Step 6: The score for each principal component was calculated;
  Step 7: A comprehensive score for all indicators was obtained.
- Establishment of indicator system and calculation of weight
  The assessment of climate change impacting suitability of potato cultivation has multiple objectives and levels. This paper combined comprehensive and hierarchical principles, relevant literature reviews^38–40,68^, expert opinions, and characteristics of potato cultivation in Jilin Province to establish an index system for evaluation of ecological environment impact, including 18 evaluation indicators: ADT ( °C d), AAT ( °C), PP (mm), SD (h), ATJ ( °C), DIF ( °C), elevation (m), slope (°), aspect (°), hill shade, sand (%), silt (%), clay (%), OM (g/kg), pH, QAK (mg/kg), AN (mg/kg), and AP (mg/kg). These indicators were classified into three categories: climatic conditions, soil environments, and topography (Table 1).
  The weight of each evaluation indicator was determined by AHP. According to relevant literatures and expert opinions, we established a judgment matrix for these evaluation indicators. Pairwise comparison was used for obtaining the relative importance score between different indicators. The consistency of pairwise importance scales is one of the important measurements for successful decision-making by AHP, which could be checked using consistency ratio (CR). If CR < 0.10, the degree of consistency is satisfactory, whereas, CR > 0.10 indicates an inconsistency^63,69^ (Table 1).
- Classification and mapping for suitability of potato cultivation

**Table 1:** Weights of all criteria used for estimating suitability of potato cultivation in the study area.

Goal	Main criteria	Weight	CR^a^	Sub-criteria^b^	Weight	CR^a^
Suitability of potato cultivation	Climate	0.53	0.001	AAT ( °C d)	0.37	0.007
				DIF ( °C )	0.24	
				ATJ ( °C )	0.17	
				ADT ( °C )	0.10	
				SD (h)	0.07	
				PP (mm)	0.05	
	Soil mechanical composition	0.18		Sand (%)	0.59	0.005
				Silt (%)	0.28	
				Clay (%)	0.13	
	Soil physico-chemical properties	0.18		pH	0.27	0.001
				OM (g/kg)	0.36	
				QAK (mg/kg)	0.17	
				AN (mg/kg)	0.12	
				AP (mg/kg)	0.08	
	Topography	0.11		Altitude (m)	0.58	0.07
				Slope (°)	0.22	
				Aspect (°)	0.10	
				Hill shade	0.10	

a CR (consistency ratio) < 0.1 means that the pairwise comparison matrix has an acceptable consistency.

b ADT: average daily temperature during the growth period ( °C); AAT: sum of active accumulated temperature ≥ 10 °C ( °C d); PP: total precipitation during the growth period (mm); SD: total sunshine duration during the growth period (h); ATJ: average temperature in July ( °C); DIF: the day/night temperature difference from July to August ( °C); OM: soil organic matter (g/kg); QAK: soil quick-acting potassium (mg/kg); AN: soil available nitrogen (mg/kg); AP: soil available phosphorus (mg/kg).

The natural breakpoint method in ArcGIS was employed to classify lands of the study area in terms of cultivation suitability. The study area was delineated into 4 zones: zone 1 (Not suitable), zone 2 (Sub-suitable), zone 3 (Suitable), and zone 4 (Most suitable) (Table 2).

**Table 2:** Dimensionless grading of evaluation values of potato cultivation suitability.

Cultivation suitability	Not suitable	Sub-suitable	Suitable	Most suitable
Evaluation value *I*	< 0.54	0.54–0.78	0.78–0.88	> 0.88

After normalizing all indicators, the cultivation suitability index was established as follows:

3 $$I={\sum }_{i}^{n}{W}_{i}{X}_{i}$$

where *I* is the suitability index for comprehensive evaluation, ${W}_{i}$ is the weight of the indicator, ${X}_{i}$ is the value after dimensionless treatment of the indicator, *i* is the comprehensive evaluation value of topography, climatic conditions, and soil environments. The larger the topography value was converted into a negative value for the calculation as the greater its value, the higher its negative impact on cultivation suitability. Meanwhile, the greater the pH value is, the more unfavorable the comprehensive evaluation of soil will be; the pH value was therefore inversed for the calculation.

#### Trends and fluctuations in changes of climatic factors and suitable areas

The fluctuations of various climatic factors over the past 58 years were analyzed by coefficient of variation (CV), which was calculated as CV = (standard deviation/mean) × 100%. Temporal trends in changes of climatic factors and suitable areas were calculated using ordinary least squares linear regression on annual data from 1961 to 2018. Among them, the trend in suitable area changes was calculated based on each grid. The significance of trends was estimated following a method that considers the temporal autocorrelation by reducing the effective sample size of the time series^70^. And the significance of temporal trends was tested at *P* < 0.1^71^.

## Results

### Inter-annual variation of climatic factors

From 1961 to 2018 (April to September), various climatic factors demonstrated noticeable changes over time in Jilin Province. Within this period, AAT (mean = 2,891.90  °C d) ranged from 2,550 °Cd to 3,150  °C d, showing a large fluctuation range (CV_1_ = 3.45%) and an obvious rising trend (*P* < 0.1) at an increasing rate of 49.27  °C d decade^−1^ (R^2^ = 0.70, *P* < 0.01). ADT (mean = 16.81  °C) was between 15.30  °C and 18.10  °C and with a small fluctuation range (CV_2_ = 2.67%), exhibiting a significant upward trend (*P* < 0.1), with an increasing rate of 0.22  °C decade^−1^ (R^2^ = 0.68, *P* < 0.01). ATJ (mean = 22.42  °C) was between 20.50  °C to 24.50  °C, with a relatively small fluctuation range (CV_3_ = 2.33%); DIF (mean = 9.77  °C) ranged from 8  °C to 11  °C, with a large fluctuation range (CV_4_ = 3.85%); however, neither of them showed a significant trend (*P* > 0.1). PP (mean = 507.37 mm) was between 380 mm and 620 mm, which fluctuated greatly (CV_5_ = 7.32%) but no significance was observed in the change trend (*P* > 0.1). SD (mean = 1,376.52 h) ranged from 1140 h to 1540 h, with a large fluctuation (CV_6_ = 3.81%) and a significant decrease (*P* < 0.1) at a rate of 25.31 h decade^−1^ (R^2^ = 0.66, *P* < 0.01) (Fig. 2). The fluctuation range of precipitation was far greater than that of temperature and sunshine (CV_5_ > CV_6_ > CV_4_ > CV_1_ > CV_2_ > CV_3_).

**Figure 3:**
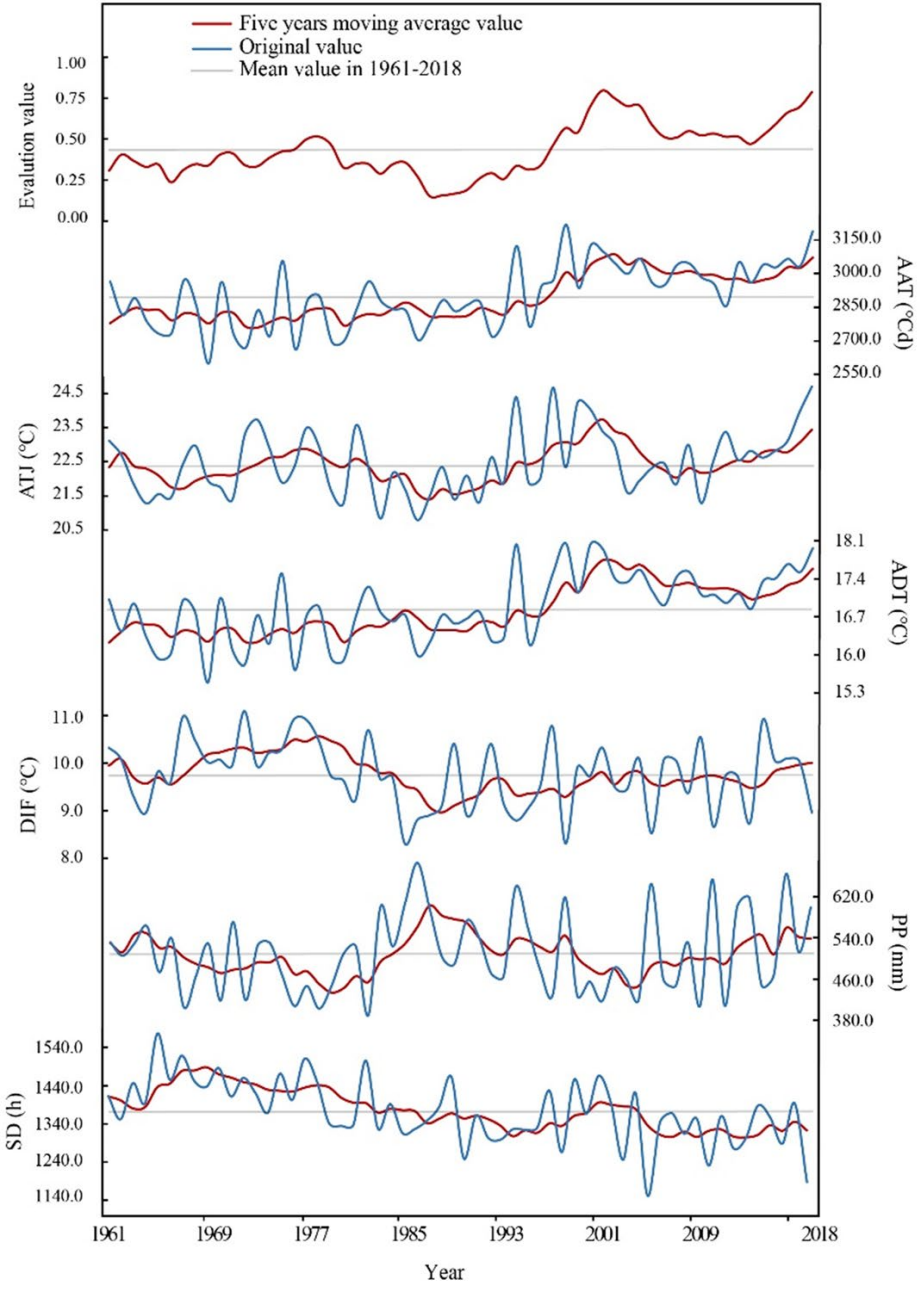
Inter-annual variation trends of climatic factors and evaluation values in the study area from 1961 to 2018. ADT: average daily temperature during the growth period (°C); AAT: sum of active accumulated temperature ≥ 10 °C ( °C d); PP: total precipitation during the growth period (mm); SD: total sunshine duration during the growth period (h); ATJ: average temperature in July ( °C); DIF: the day/night temperature difference from July to August ( °C).

The comprehensive evaluation value (mean = 0.43) of climatic suitability for potato cultivation in Jilin Province fluctuated greatly (CV = 37.05%). Splitting the period of 1961–2018 into three distinct intervals was achieved by inspecting the linear trend of comprehensive values. It was found that the value of climatic suitability fluctuated slightly from 1961 to 1989 (CV = 24.06%) and showed no significance in change trend (*P* > 0.1). From 1990 to 2001, it fluctuated greatly (CV = 49.04%) and presented a steep upward trend (*P* < 0.05). From 2002 to 2018, the fluctuation was small (CV = 16.68%), which decreased slowly at first and then increased (Fig. 3).

### Climate inclination rates varied across the study area

**Figure 4:**
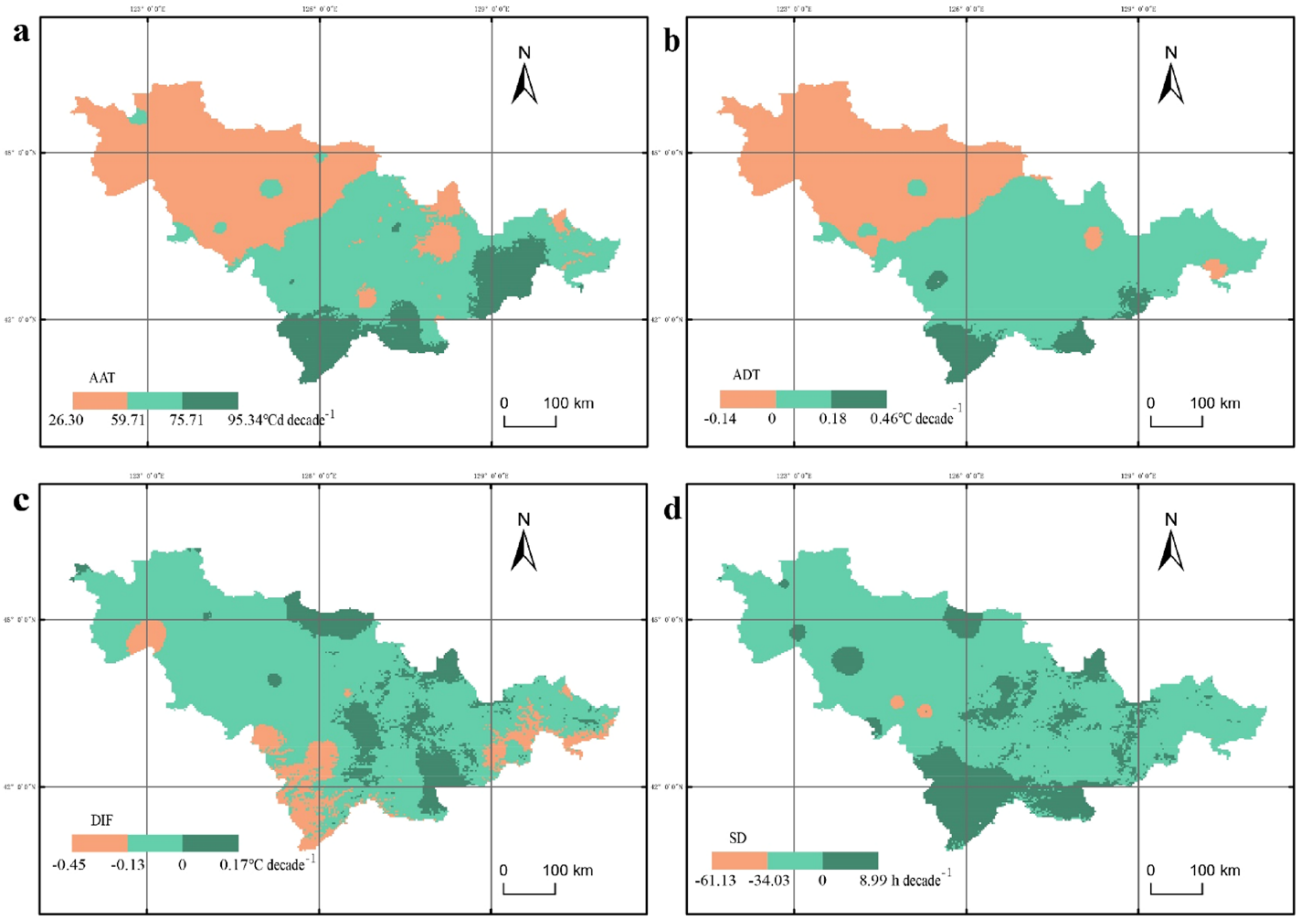
Maps for spatial distribution of climate inclination rates varied across Jilin Province from 1961 to 2018. (**a**) The active accumulated temperature of ≥ 10 °C. (**b**) Average daily temperature during the growth period. (**c**) The average day/night temperature difference from July to August. (**d**) Sunshine durations. For visualization, maps were built using ArcGIS v. 10.4.1 (http://www.esri.com/software/arcgis).

During the period of 1961–2018, within Jilin Province, the values of both sunshine and temperature were greater in the western plains than in the eastern mountainous regions while the precipitation gradually decreased from the southeast to the northwest. AAT ranged from 910 to 3,390  °C d in the study area, which showed an overall increasing trend, with the tendency rate gradually decreased from the west to the east (Fig. 4a). The area of regions with an AAT tendency rate between 75.71–95.34, 59.71–75.71, and 26.30–59.71  °C d decade^−1^ accounted for 44.54%, 40.68%, and 14.78% of the total area, respectively. There were 96.96% area of Jilin Province passed the significance test (*P* < 0.1; Fig.S1a). ADT was between 6.5–24.5  °C, showing an increasing trend and accounting for 93.36% of the total area (Fig. 4b). The tendency rate of ADT was greater in the northwest than in the southeast. The area of regions with an ADT tendency rate between 0–0.18, 0.18–0.46, and − 0.14–0 °C decade^−1^ accounted for 52.85%, 40.50%, and 6.64% of the total area, respectively. There were 50.99% area of Jilin Province passed the significance test (*P* < 0.1; Fig.S1b). DIF was between 7.0–16.0  °C in Jilin Province, which showed a decreasing trend in 88.71% of the regions, with a climate trend slope range of − 0.45–0 °C decade^−1^. The rest of the study area possessed a DIF tendency rate of 0–0.17  °C decade^−1^ (Fig. 4c). There were 20.25% area of the study area passed the significance test (*P* < 0.1; Fig.S1c). SD was in the range of 1035–1725 h, showing a significant downward trend in most of the study area (Fig. 4d). The area of regions with a SD tendency rate between − 34.03–0 and − (61.13–34.03) h decade^−1^ accounted for 77.77% and 21.76% of the total area. There were 76.04% area of Jilin Province passed the significance test (*P* < 0.1; Fig.S1e). However, ATJ was between 14.5–25.5  °C and PP was between 350–1,020 mm. The tendency rates of both ATJ and PP showed no significance in inter-annual changes (*P* > 0.1; Fig.S1d, f) during 1961–2018.

### Trends in climate-driven suitability zonation varied across the study area

**Figure 5:**
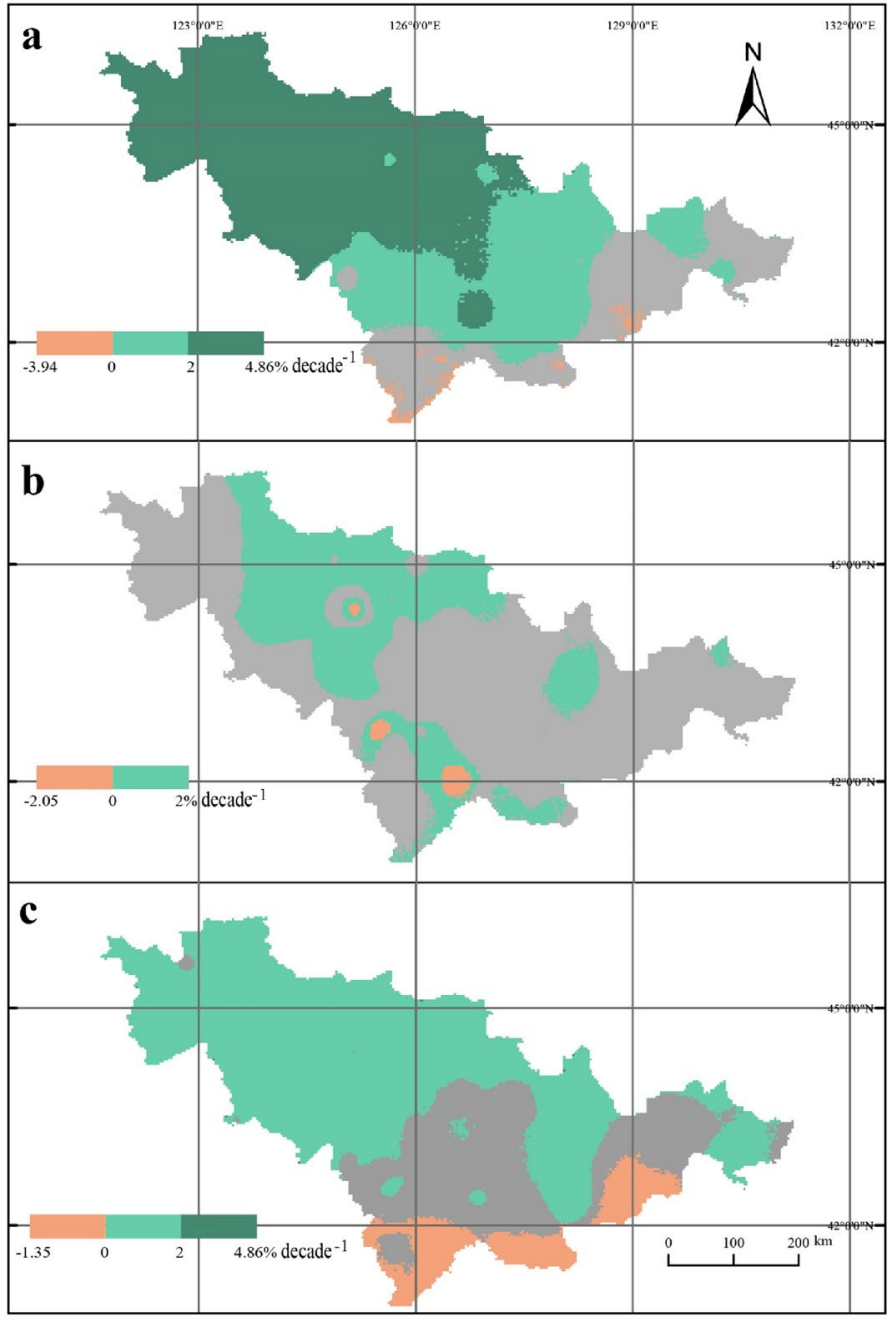
Maps for trends in climate-driven suitability zonation across Jilin Province from 1957 to 2018. (**a**) 1961–1988; (**b**) 1988–2018; (**c**) 1961–2018. The grey shades in (**a**–**c**) indicate the regions where trends in area change of suitability zonation failed the significance test (P > 0.1). For visualization, maps were built using ArcGIS v. 10.4.1 (http://www.esri.com/software/arcgis).

From 1961 to 1988, evident changes were observed in the suitable areas for potato cultivation, of which approximately 80.99% showed significance (Fig. 5a). The results showed that 1.21% area of Jilin Province suitable for potato cultivation were mainly distributed in the marginal zone to the southeast, with a negative growth trend (− 3.94%–0 decade^−1^, *P* < 0.1). The 28.52% area of Jilin Province suitable for potato cultivation were mainly located in the central and eastern parts, showing a slow growth trend (0–2% decade^−1^, *P* < 0.1). The 51.27% area of Jilin Province suitable for potato cultivation occurred mainly in the northwestern regions, exhibiting a significant increasing trend (2%–4.86% decade^−1^, *P* < 0.01).

From 1988 to 2018, the degree of changes in suitable areas was relatively small, and 34.26% of the areas passed the significance test (Fig. 5b). Our results show that 7.36% area of Jilin Province suitable for potato cultivation were mainly distributed in the southeastern marginal zone, showing a slow negative growth trend (− 2.05%–0 decade^−1^, *P* < 0.1). The 26.90% area of Jilin Province showing a slow increasing trend (0–1.77% decade^−1^, *P* < 0.1) were distributed in the northwest.

During the period of 1961–2018, the suitable areas for potato cultivation in Jilin Province changed significantly. About 72.36% of the areas passed the significance test (Fig. 5c). We found that 9.78% area of Jilin Province suitable for potato cultivation were mainly distributed in the marginal zone to the southeast, showing a slow negative growth trend (− 1.35%–0 decade^−1^, *P* < 0.1). The 30.63% area of Jilin Province suitable for potato cultivation were mainly located in the central and eastern regions, with a slow growth trend (0–1% decade^−1^, *P* < 0.1). The potato cultivation suitability showing a relatively rapid growth trend in 31.95% (1–2% decade^−1^, *P* < 0.01) and 0.03% (2–4.86% decade^−1^, *P* < 0.01) area of Jilin Province, respectively, mainly occurred in the northwestern regions.

### Temporal and spatial distributions of suitable areas for potato cultivation

**Figure 6:**
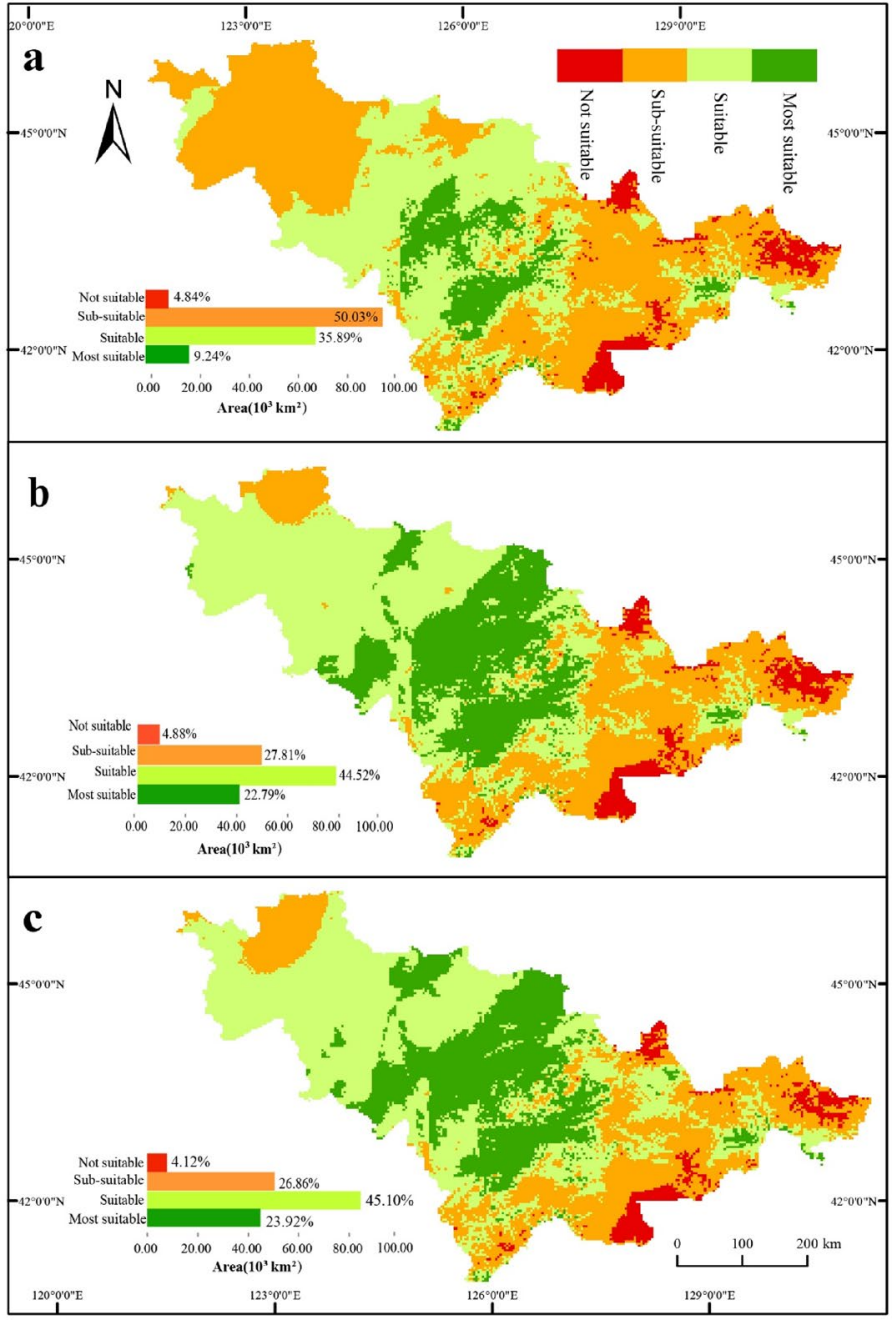
Maps for spatio-temporal changes in the distribution of suitability zonation for potato cultivation in Jilin Province. (**a**) In 1961, the areas of “Not suitable”, “Sub-suitable”, “Suitable”, and “Most suitable” areas were 9.08 × 10^3^, 93.76 × 10^3^, 67.25 × 10^3^, and 17.31 × 10^3^ km^2^, respectively. (**b**) In 1988, the areas of “Not suitable”, “Sub-suitable”, “Suitable”, and “Most suitable” areas were 9.15 × 10^3^, 52.11 × 10^3^, 83.42 × 10^3^, and 42.71 × 10^3^ km^2^, respectively. (**c**) In 2018, the areas of “Not suitable”, “Sub-suitable”, “Suitable”, and “Most suitable” areas were 7.72 × 10^3^, 50.33 × 10^3^, 84.53 × 10^3^, and 44.82 × 10^3^ km^2^, respectively. For visualization, maps were built using Esri ArcGIS v. 10.4.1 (http://www.esri.com/software/arcgis).

From the perspective of spatial distribution, the “Most suitable” and “Suitable” areas exhibited a trend towards northwest, while the “Sub-suitable” area tended to shrink to the eastern mountainous areas. According to Fig. 3, the comprehensive evaluation value in 1988 was the lowest during the past 60 years. Therefore, the three periods, including 1961–2018, 1961–1988, and 1988–2018, were selected to compare the changes in the suitable areas (Fig. 6a-c). The area of “Most suitable” areas increased by 25.40 × 1,000 km^2^ (13.5%) from 1961 to 1988 (Fig. 6a,b) and by 2.11 × 1,000 km^2^ (1.13%) from 1988 to 2018 (Fig. 6b,c), with a 12.42% decrease in the percentage increase in area. The area of “Suitable” areas increased by 16.17 × 1,000 km^2^ (8.63%) from 1961 to 1988 (Fig. 6a,b) and by 1.10 × 1,000 km^2^ (0.59%) from 1988 to 2018 (Fig. 6b,c), with a 8.04% decrease in the percentage increase in area. The area of “Sub-suitable” areas decreased by 41.65 × 1,000 km^2^ (22.23%) from 1961 to 1988 (Fig. 6a,b) and by 1.77 × 1,000 km^2^ (0.95%) from 1988 to 2018 (Fig. 6b,c), with a 21.28% decreased in the area ratio. The area of “Not suitable” areas increased by 0.08 × 1,000 km^2^ from 1961 to 1988 (Fig. 6a,b) and decreased by 1.43 × 1,000 km^2^ from 1988 to 2018 (Fig.6b,c), with little change in the study area.

In general, from 1961 to 2018 (Fig. 6a–c), the “Most suitable” areas were mainly distributed in the mountainous areas of the central and eastern regions of Jilin Province, where the climatic conditions (see Fig.S2-S4) were conducive to potato tuber growth and starch accumulation. The “Suitable” areas were mainly distributed in the central and western plains, where the climatic conditions were suitable for potato cultivation and growth. The “Sub-suitable” areas were mainly distributed in low-altitude mountainous areas in the east, where precipitation conditions (see Fig.S2f, S3f, S4f) were favorable for potato tuber enlargement, and the overall climatic conditions were suitable for potato growth. The “Not suitable” areas were located in the eastern high altitudes, where the distributions of water, heat, and light resources were uneven and the heat is insufficient; the overall climatic conditions were not suitable for potato tuber growth.

### Variation in the area of suitability zonation for potato cultivation

**Figure 7:**
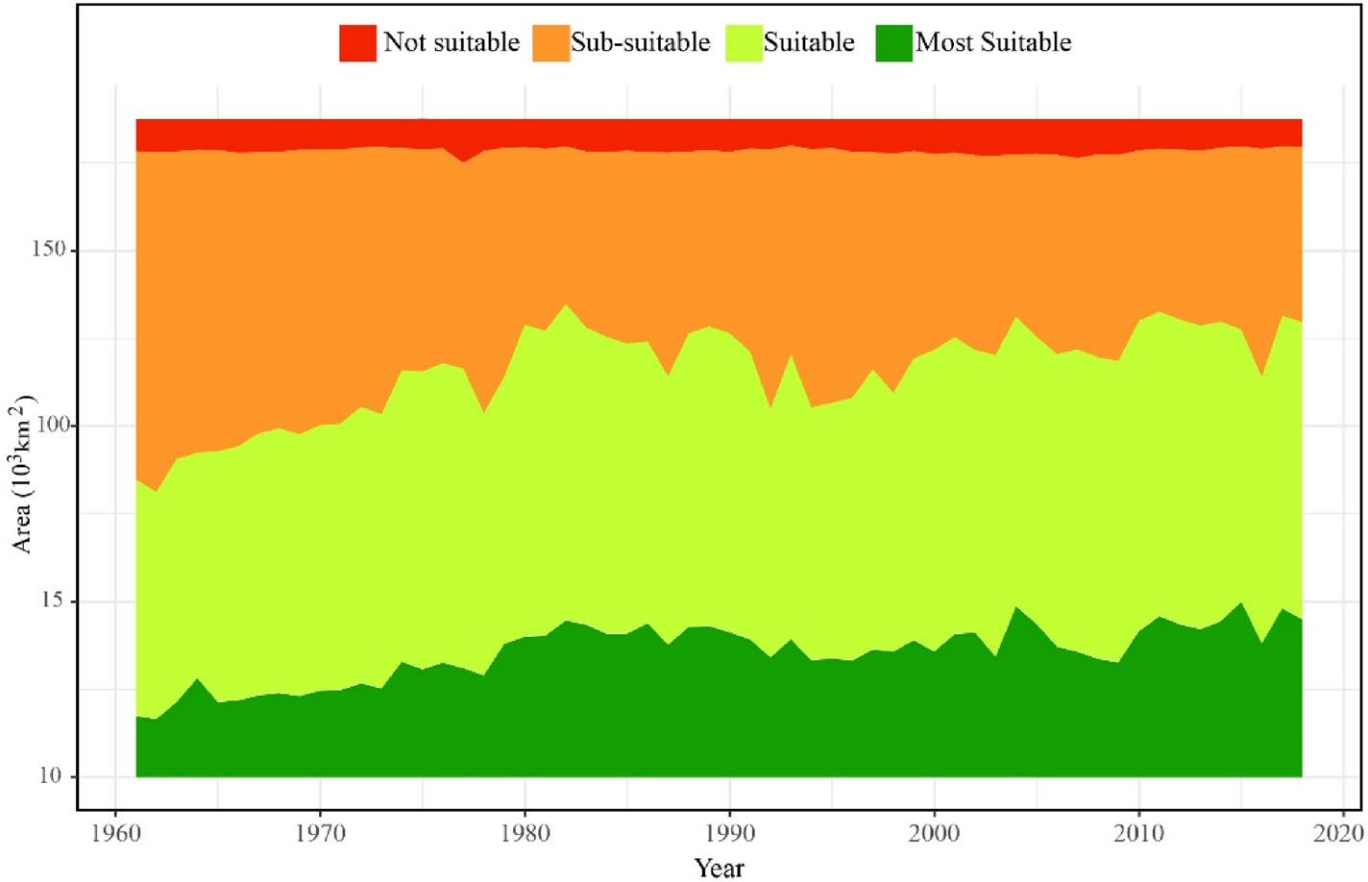
Changes of suitable areas for potato cultivation in Jilin Province.

The suitable areas for potato cultivation in Jilin Province from 1961 to 2018 were analyzed annually, and the annual change of suitable areas was obtained (Fig. 7).

We found that the maximum area of “Most suitable” areas was 4.94 × 1,000 km^2^ in 2015, and the minimum was 16.50 × 1,000 km^2^ in 1962. The area of “Most suitable” areas increased significantly (*P* < 0.01), with an increasing rate of 0.37 × 1,000 km^2^ decade^−1^ (R^2^ = 0.58) during the past 60 years. From the 1960s to the late 1970s, the area of “Most suitable” areas increased significantly (*P* < 0.0001), with an increasing rate of 0.85 × 1,000 km^2^ decade^−1^ (R^2^ = 0.76). From the early 1980s to the mid-1990s, the area exhibited a significant downward trend (*P* < 0.001), with a decreasing rate of 0.54 × 1,000 km^2^ decade^−1^ (R^2^ = 0.51). From the late 1990s to 2018, the area of “Most suitable” areas increased slowly (*P* < 0.05), with an increasing rate of 0.40 × 1,000 km^2^ decade^−1^ (R^2^ = 0.26).

The area of “Suitable” areas showed a significant increasing trend (*P* < 0.1), with a maximum value of 90.00 × 1,000 km^2^ in 1982, a minimum value of 64.05 × 1,000 km^2^ in 1964, and an increasing rate over the past 60 years of 0.20 × 1,000 km^2^ decade^−1^ (R^2^ = 0.28). From the 1960s to the late 1970s, the area of “Suitable” areas increased significantly (*P* < 0.05), with an increasing rate of 0.92 × 1,000 km^2^ decade^−1^ (R^2^ = 0.66). From the early 1980s to the mid-1990s, the area of “Sub-suitable” areas showed a sharp downward trend (*P* < 0.0001) at a rate of 0.92 × 1,000 km^2^ decade^−1^ (R^2^ = 0.61). From the late 1990s to 2018, there was no significant change in the area of “Suitable” areas (*P* > 0.1).

The area of “Sub-suitable” areas showed a significant decline (*P* < 0.05) at a rate of 0.58 × 1,000 km^2^ decade^−1^ (R^2^ = 0.53) in the past 60 years. Its maximum value was 97.17 × 1,000 km^2^ in 1962, and minimum value was 45.13 × 1,000 km^2^ in 1982. From the 1960s to the late 1970s, there was a significant increase in the area of “Sub-suitable” areas (*P* < 0.01), with an increasing rate of 1.77 × 1,000 km^2^ decade^−1^ (R^2^ = 0.83). From the early 1980s to the mid-1990s, the area of “Sub-suitable” areas in Jilin Province increased significantly (*P* < 0.01), with an increasing rate of 1.45 × 1,000 km^2^ decade^−1^ (R^2^ = 0.62); while from the late 1990s to 2018, it slowly decreased (*P* < 0.1) at a rate of 0.43 × 1,000 km^2^ decade^−1^ (R^2^ = 0.22).

There was no significant change in the area of “Not suitable” areas. The maximum was 12.43 × 1,000 km^2^ in 2015, and the minimum was 7.44 × 1,000 km^2^ in 1962 (*P* > 0.1).

Overall, the proportion of the suitable areas for potato cultivation in Jilin Province from 1961 to 2018 was “Suitable” areas > “Sub-suitable” areas > “Most suitable” areas > “Not suitable” areas. Among them, the average values of “Most suitable”, “Suitable”, “Sub-suitable”, and “Not suitable” areas were 35.42 × 1,000, 79.91×1,000, 63.10 × 1,000, and 8.97 × 1,000 km^2^, respectively. In the past 58 years, the area of “Most suitable” areas for potato cultivation has increased more sharply than that of “Suitable” areas, and both of them showed a significant decline from the early 1980s to the 1990s, with a more significant decrease observed in the area of “Suitable” areas. However, the change in the area of “Sub-suitable” areas showed an opposite trend to that of the aforementioned two.

## Discussion and conclusion

### Discussion

Results obtained from this study show that the increase in the suitability of potato cultivation is more significant in the plains of northwestern Jilin Province than in the mountainous areas of the eastern part. From the perspective of time series comparison, the speed of northward migration and expansion of suitable areas in the later period (1961–1990) was significantly slower than that in the earlier period (1991–2018) (Fig. 5). The significant increase in temperature of semi-arid regions of the northern low-altitude plains has a great impact on the increase in the suitability of potato cultivation, whereas the influence of warm temperature on the suitability of potato cultivation was relatively small in the eastern mountainous areas near the sea (Fig. 6). We found that climate change has expanded the extent of suitable areas for potato cultivation. During the potato growth period (April to September) of 1961–2018 in Jilin Province, AAT showed a significant upward trend, SD showed a downward trend, and PP fluctuated to a large extent without showing an obvious trend. Under the influence of global climate change, the suitable areas for potato cultivation in Jilin Province have been expanding, indicating that the increase in temperature has had a positive impact on the suitability of potato cultivation in Jilin Province over the years, which is consistent with the trend of climatic suitability for potato cultivation^39,72^.

Through the comparative analysis of the distribution of suitable areas for potato cultivation and the dominant potato production areas^49^ in Jilin Province, we found that the current main potato production areas in Jilin Province are mainly included in the “Most suitable” areas, distributed in the center of Jilin Province. This suggests that, more accurate results of suitability zoning were obtained from the current research compared with previous studies on climatic suitability^39,52^ due to the addition of soil factors. The result generated using the evaluation method in this paper, to some extent, can reflect the distribution of suitable areas for potato cultivation.

In addition, it is worth mentioning that not only climate change but also the potato variety, soil fertility, farming system, and production technology^5,21^ can influence potato growth and development. Further research should consider possible climate change scenarios in the future, in combination with field conditions, irrigation technologies, and other modern measures, to provide a more comprehensive reference for potato cultivation management in Jilin Province. We found that once average daily temperature during the growth period in the study area passed the threshold (~16.5 °C), on a large scale, its effect on the total suitable areas for potato cultivation diminished. In the earlier period (1961–1990), the temperature increased at a relatively low rate, but the mapped areas under the classes “Most Suitable” and “Suitable” increased enormously. In the later period (1991–2018), the temperature increased faster, however, the changes in the generally suitable areas were smaller. In conclusion, it showed that changes in the area of suitable potato cultivation in Jilin Province are sensitive to the temperature variation during the earlier period. Meanwhile, since potatoes have certain tolerance to temperature and rainfall, as the temperature gets closer and closer to the threshold, the suitability of potato cultivation decreases. When the temperature exceeds the suitable temperate range for potato growth, potato cultivation may be negatively affected by climate change.

Comparing the temporal trends of actual production areas with calculated suitable areas, we found, it was consistent for the estimated and actual trends of suitable potato cultivation areas. Farmer already adapted to climate change through shifting the potato plantation from unsuitable to suitable regions. Referring to the Jilin Province Agricultural Statistics Yearbook^73^, it was found that during 1984–2018, from west to east, the planting areas of potato were 51.94km^2^ (7.26%), 266.98km^2^ (36.74%), 55.60km^2^ (7.69%), 69.17km^2^ (9.58%), 146.63km^2^ (19.69%), 30.83km^2^ (4.25%), 51.15km^2^ (7.21%), 13.50km^2^ (1.89%), and 41.54km^2^ (5.82%) in Baicheng, Songyuan, Changchun, Jilin City, Siping, Liaoyuan, Tonghua, Baishan, and Yanbian Autonomous Prefecture, respectively. The average yields were 4,308.04 kg/hm^2^, 6,046.84 kg/hm^2^, 6,076.32 kg/hm^2^, 5,940.79 kg/hm^2^, 5,696.14kg/hm^2^, 6,525.99 kg/hm^2^, 5,220.18kg/hm^2^, 4,576.95 kg/hm^2^, and 3,871.02 kg/hm^2^ in Baicheng, Songyuan, Changchun, Jilin City, Siping, Liaoyuan, Tonghua, Baishan, and Yanbian Autonomous Prefecture, respectively. The actual plantation areas of potato, as urbanization and industrialization, gradually decreased from 770.66 km^2^ to 359.04 km^2^ (Fig.S5) with slight decrease in the eastern alpine mountainous, and obvious decreases in the central regions although annual fluctuation. In recent years, the weight of potato cultivation area in all agriculture land usage has shown the upward trend (Fig.S6). Meanwhile, the potato yield per unit area were gradually enhanced with the greatest increases in the central (e.g., Changchun and Songyuan) and western (e.g., Liaoyuan and Baishan) regions of Jilin Province (Fig. S7), which were the “Most suitable” and “Suitable” areas, respectively. Consequently, the conclusion in this study can truthfully reflect the current cultivation of potato in Jilin Province. From the perspective of geographical distribution, the suitable areas gradually expand to the plains in the northwestern Jilin Province: (i) the sandy soil of the northern region is conducive to the cultivation and production of commercial potatoes; (ii) the mature drip irrigation technology is beneficial to resist adverse effects of increased evaporation caused by future temperature increase; (iii) the transportation condition is convenient and advantageous to the commodity potato transport. Therefore, it is recommended that the future potato industry chain be situated in the northern region of Jilin Province to form a large-scale potato industrial cluster.

### Conclusion

In the current study, the impact of climate change on the spatial distribution of suitable areas for potato cultivation was analyzed, and the temporal and spatial variations of this distribution from 1961 to 2018 were explored. The suitability of potato cultivation in Jilin Province was assessed on the basis of a comprehensive set of criteria associated with the multi-criterion, decision-based AHP-PCA approach. Our results show that the average values of AAT, PP, and SD changed greatly, leading to a change in the climatic suitability of potato production in Jilin Province. Suitable areas for potato cultivation changed little in the middle- and high-altitude mountainous areas in the east, but significant changes in suitable areas were observed in the low-altitude plains in the central and western regions.

From the perspective of spatial distribution, the “Most suitable” areas for potato cultivation are concentrated in the central region of Jilin Province, the “Suitable” areas are distributed in the plains in the northwestern part, the “Sub-suitable” areas are mainly located in the eastern mountainous areas, and the “Not suitable” areas occur in the eastern high altitudes. From the perspective of spatio-temporal changes, the impact of key climatic factors on potato cultivation has changed from 1961 to 2018: (i) the climatic suitability of potato cultivation moved northward; (ii) the “Most suitable” and “Suitable” areas for potato cultivation expanded, but the distribution of “Sub-suitable” areas narrowed down.

The area of “Most suitable” areas expanded by 27.51 × 1,000 km^2^, and that of “Suitable” areas expanded by 17.27 × 1,000 km^2^, suggesting that climate change has had a favorable impact on potato cultivation in Jilin Province in the past decades. This research conducted an in-depth analysis of the climate change-induced regional changes in suitable areas for potato production in Jilin Province from a spatio-temporal perspective and provided solid support for potato cultivation to adapt to the new climatic conditions.

## Supplementary Information

Supplementary Information.
