# Supplementary material for: Impacts of climate change on suitability zonation for potato cultivation in Jilin Province, Northeast China
Source: Sci Rep. 2021 Sep 6;11:13103. doi: 10.1038/s41598-021-91273-5 (PMC8421430; doi:10.1038/s41598-021-91273-5)
Supplement: Supplementary file 1 — Supplementary Information. [file 41598_2021_91273_MOESM1_ESM.pdf]

# Impacts of climate change on suitability zonation for potato cultivation in Jilin Province, Northeast China

**Yaqiu Zhu[1], Qiang Yu[1,*], Qiyou Luo[1], Hua Zhang[1], Jinling Zhao[2], Zhanghong Ju[1], Yating Du[1] &Yadong Yang[1,*]**

[1]Institute of Agricultural Resources and Regional Planning, Chinese Academy of Agricultural Sciences, Beijing, 100081, China.
[2]State Key Laboratory of Vegetation and Environmental Change, Institute of Botany, Chinese Academy of Sciences, Beijing, 100093, China.
* email: yuqiang@caas.cn; yangyadong@caas.cn

## Supplementary information is available for this paper

**Table S1.** Statistical characteristics of soil mechanical compositions in Jilin Province

| Type | Number | Max/% | Min/% | Mean/% | Median | Standard deviation | Skewness | K-S test | CV/% |
|---|---|---|---|---|---|---|---|---|---|
| Sand | 81 | 86.62 | 0.00 | 46.31 | 47.39 | 21.43 | -0.40 | 0.11 | 46.28 |
| Silt | 81 | 51.57 | 2.75 | 26.35 | 26.64 | 13.86 | 0.02 | -1.12 | 52.62 |
| Clay | 81 | 54.86 | 7.32 | 23.35 | 23.28 | 9.32 | 0.79 | 1.39 | 39.93 |

**Table S2.** Statistical characteristics of soil physico-chemical properties in Jilin Province

| Type | Number | Mean | Median | Standard deviation | Skewness | Mean | Median | S-W test | Sig. |
|---|---|---|---|---|---|---|---|---|---|
| PH | 79 | 6.32 | 5.82 | 1.28 | -1.06 | 0.57 | 20.29 | 0.86 | 0.000 |
| OM (g/kg) | 79 | 32.92 | 30.98 | 12.34 | 0.64 | 0.74 | 37.48 | 0.98 | 0.255 |
| AP (mg/kg) | 79 | 50.18 | 28.15 | 49.14 | 2.13 | 1.56 | 97.93 | 0.82 | 0.000 |
| QAK (mg/kg) | 79 | 144.06 | 134.40 | 70.01 | 3.23 | 1.45 | 48.60 | 0.86 | 0.000 |
| AN (mg/kg) | 79 | 160.03 | 146.27 | 76.29 | 1.36 | 0.96 | 47.68 | 0.94 | 0.003 |

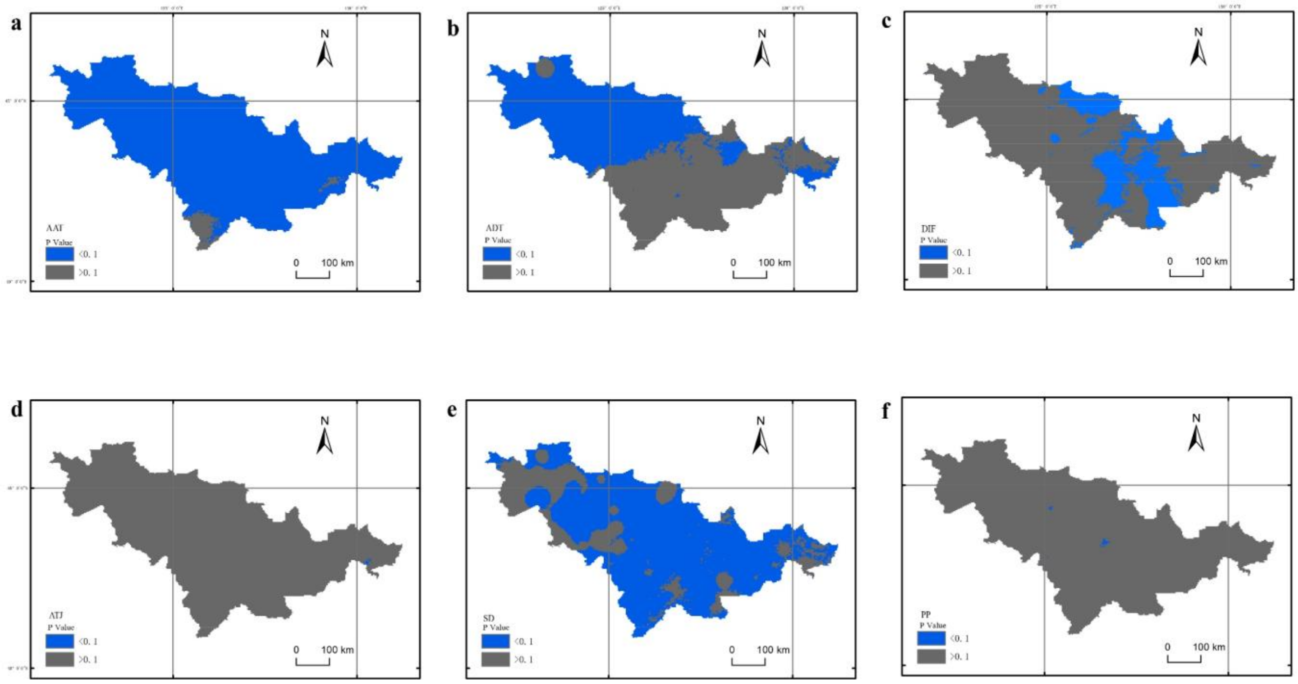

**Figure S1.** Results of *t* test for climate inclination rates of the six climatic factors from 1961 to 2018. The grey shades in (a–f) indicate the regions where trends in change of climatic factors were not significant ($P > 0.1$). (a) AAT: sum of active accumulated temperature ≥ 10°C; (b) ADT: average daily temperature during the growth period; (c) DIF: the day/night temperature difference from July to August; (d) ATJ: average temperature in July; (e) SD: total sunshine duration during the growth period; (f) PP: total precipitation during the growth period.

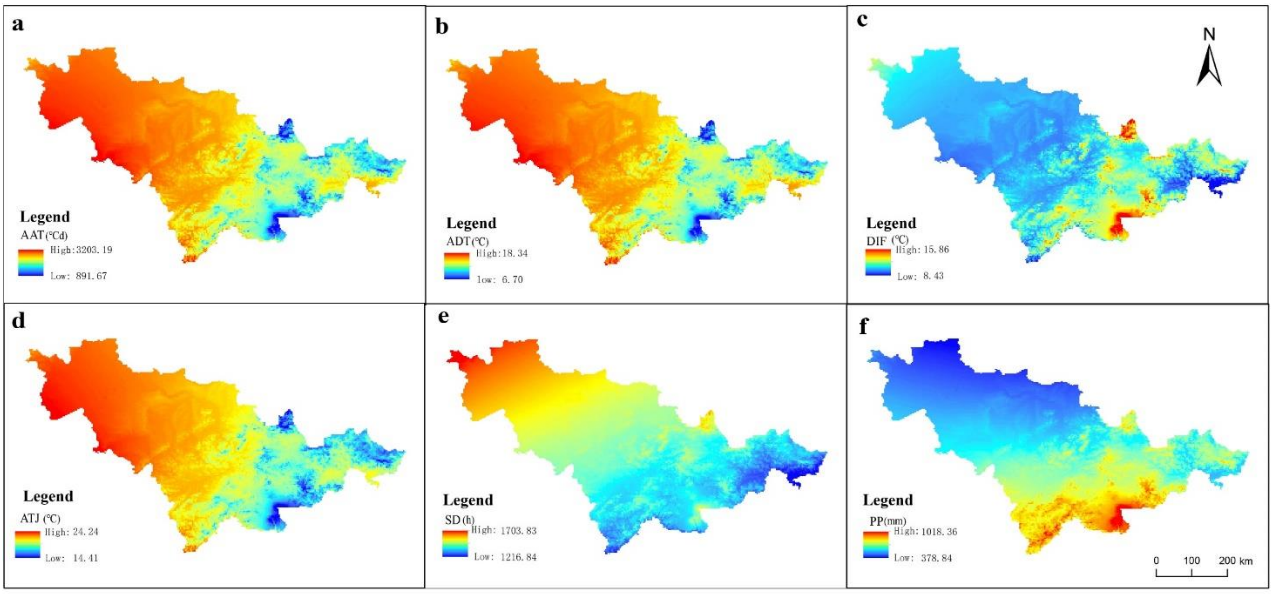


**Figure S2.** Spatial distribution of the six climatic factors in 1961. (a) AAT: sum of active accumulated temperature ≥ 10°C; (b) ADT: average daily temperature during the growth period; (c) DIF: the day/night temperature difference from July to August; (d) ATJ: average temperature in July; (e) SD: total sunshine duration during the growth period; (f) PP: total precipitation during the growth period (same below).

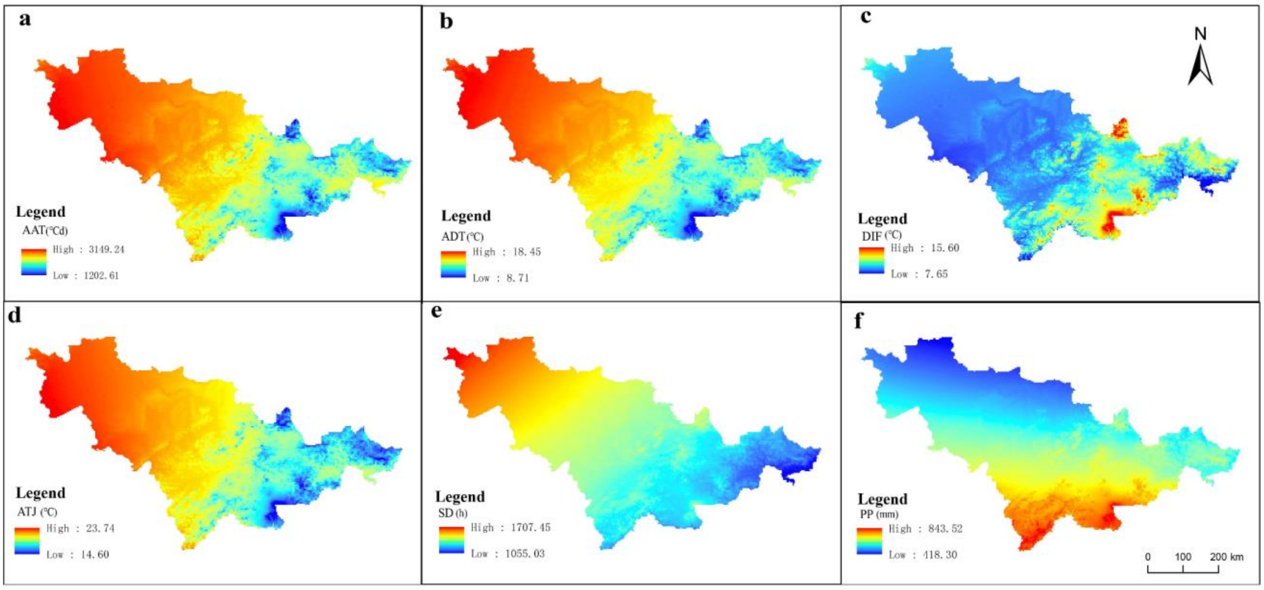


**Figure S3.** Spatial distribution of the six climatic factors in 1988.

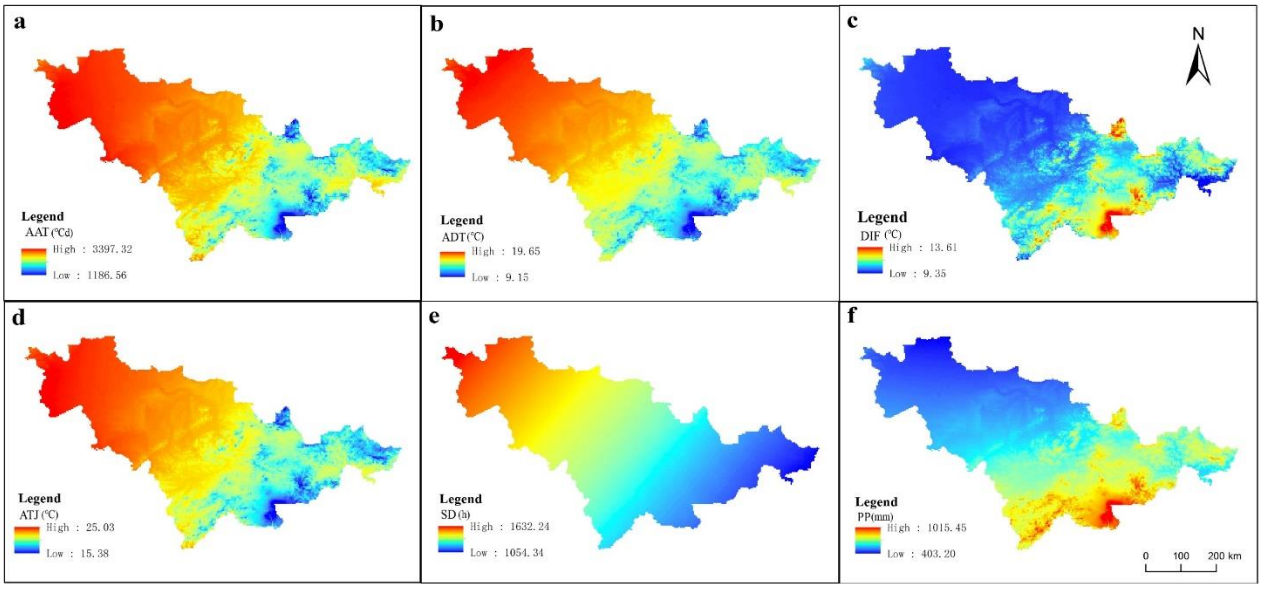


**Figure S4.** Spatial distribution of the six climatic factors in 2018.

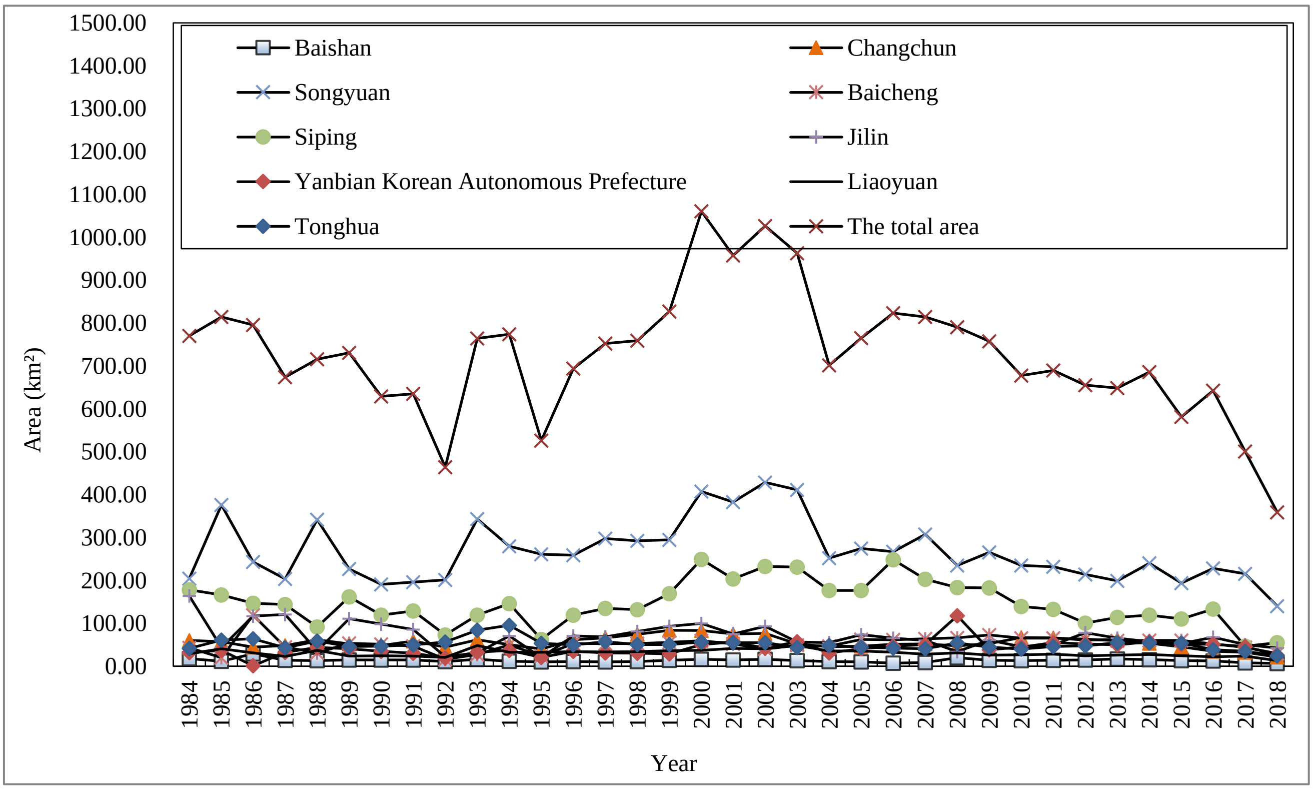


**Figure S5.** Changes of potato cultivation area in Jilin Province from 1984 to 2018.

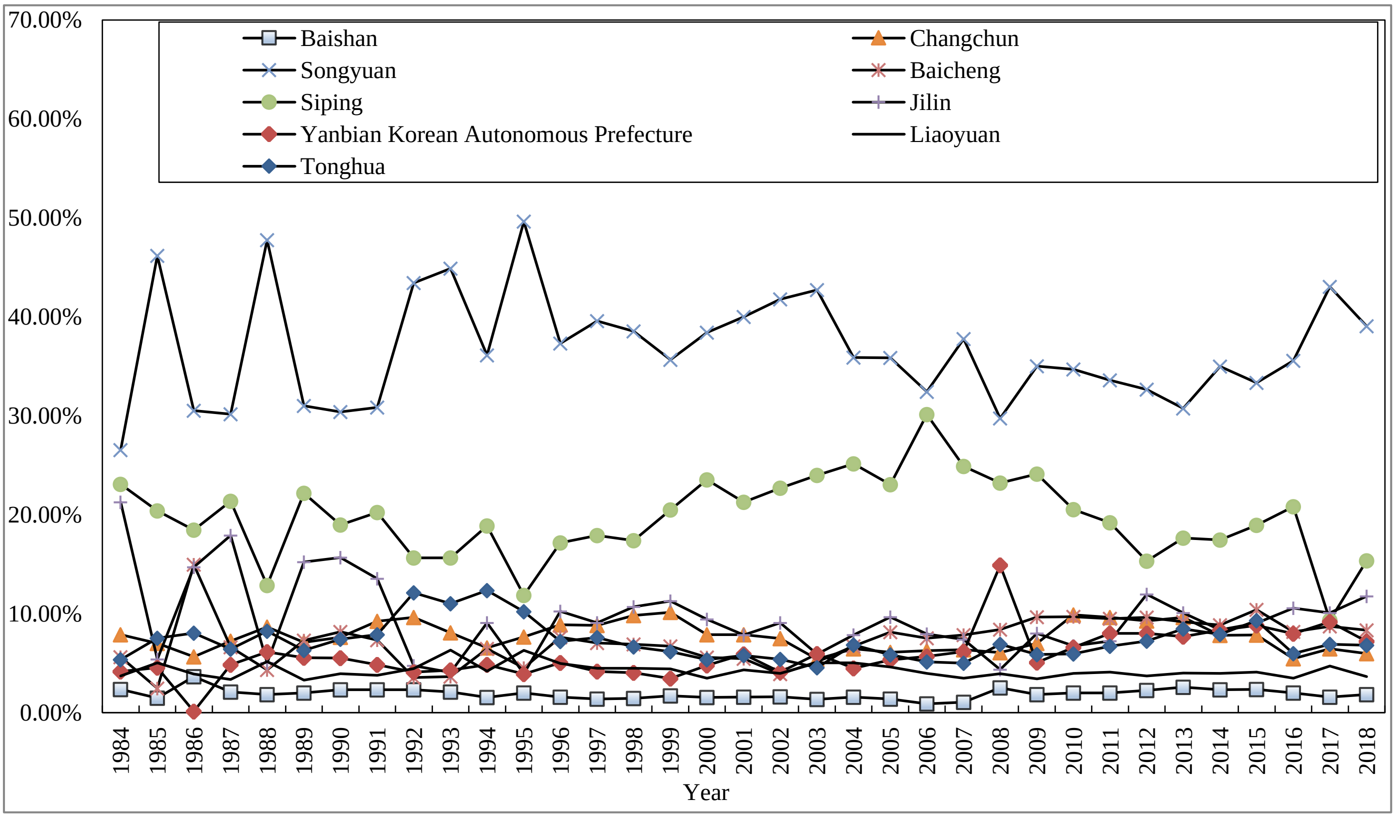


**Figure S6.** Changes in the proportion of potato cultivation area in Jilin Province from 1984 to 2018

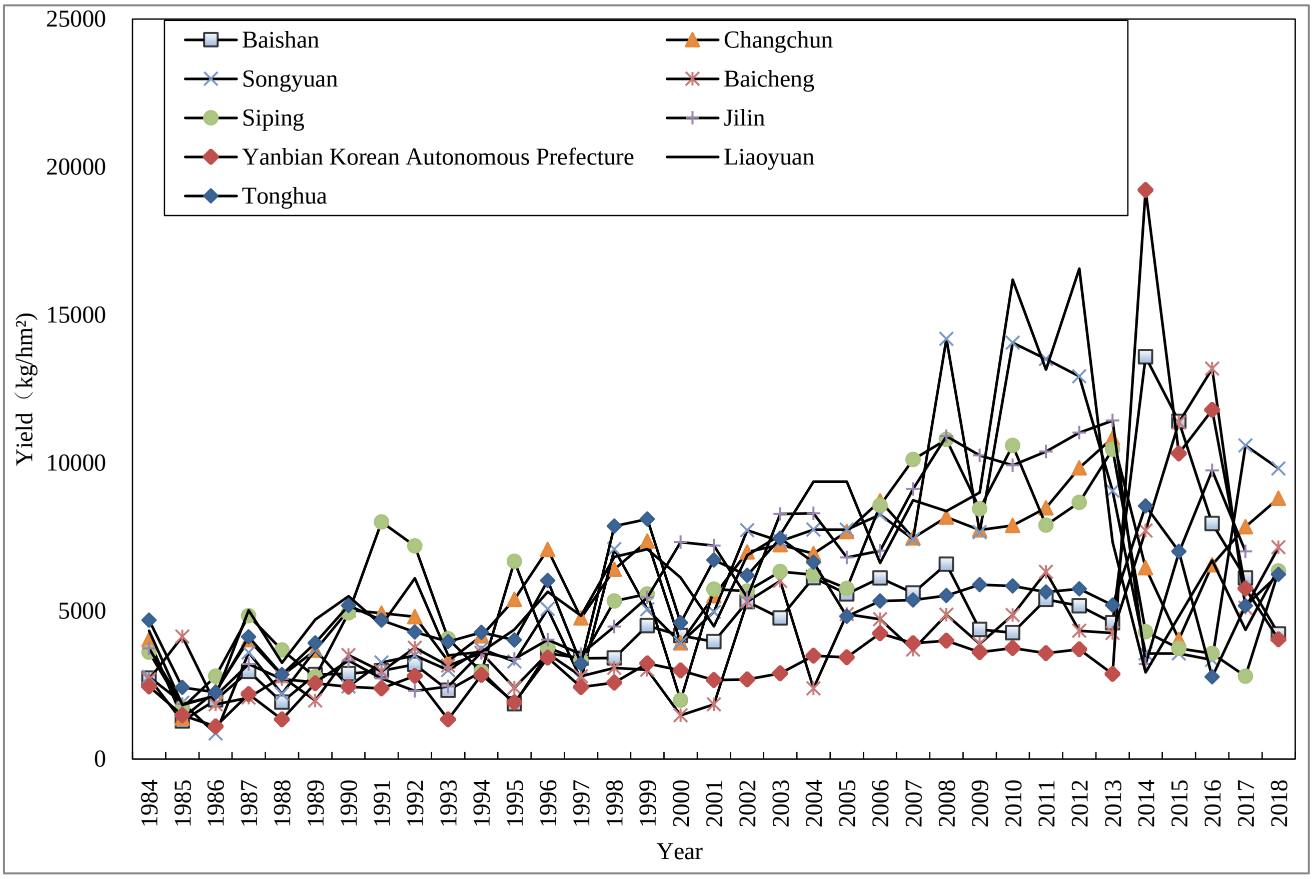


**Figure S7.** Changes of potato yield per unit area in Jilin Province from 1984 to 2018.
